# Dish swap across a weekly menu can deliver health and sustainability gains

**DOI:** 10.1038/s43016-025-01218-8

**Published:** 2025-08-11

**Authors:** Annika N. Flynn, Taro Takahashi, Alex Sim, Jeffrey M. Brunstrom

**Affiliations:** 1 Nutrition and Behaviour Unit, School of Psychological Science, University of Bristol, Bristol, UK; 2 Bristol Veterinary School, University of Bristol, Bristol, UK; 3 Agri-Food and Biosciences Institute, Hillsborough, UK; 4 Catering Operations, University of Bristol, Bristol, UK; 5 National Institute for Health and Care Research Bristol Biomedical Research Centre and the National Institute for Health and Care Research Applied Research Collaboration West (NIHR ARC West), Bristol, UK

**Keywords:** Human behaviour, Psychology and behaviour

## Abstract

A weekly canteen menu comprising 15 dishes (3 dishes × 5 days) has 1.4 million unique configurations. Here food choice was monitored over four weeks ( ~ 5,000 meals) in a UK university residence. Without students noticing, mathematically optimized menus achieved 30.7% and 6.3% reductions in carbon footprint and saturated fatty acid intake, respectively. This demonstrates the potential of strategic menu manipulation to benefit health and the environment, without the need for recipe changes.

## Abstract (web summary)

Menu manipulation is a promising strategy to increase environmental and health benefits of food choices. Drawing on data from a UK university residence, this study illustrates such potential.

## Main

Many population-level approaches to demand-side behaviour change fall under one of four broad categories: they educate and inform^1^, they change choice architecture within a single event^1,2^, they restrict choice options through regulatory measures (for example, legal minimum age to purchase alcohol) or they induce change through (dis-)incentive-based measures (for example, Soft Drinks Industry Levy^3^). Many countries educate youth about the health risks of smoking and excessive alcohol consumption^4,5^, and they combine this with warning labels^6,7^ and legislation that limits access to certain age groups^8^. The same approaches are also used to improve our diet; for example, the UK National Health Service advises consuming five servings of fruit or vegetables a day^9^.

Nonetheless, compared to habits related to alcohol or nicotine, the challenge of modifying dietary behaviour is more complex for two reasons. First, food is essential (nutrients and calories must be consumed to sustain life), meaning that the goal is not simply to encourage the rejection of food but to shift food choices to more desirable alternatives. Second, our gut capacity is finite and, therefore, our decision to consume a certain meal necessarily means that other options cannot be consumed. Recognizing these principles, we see a hidden opportunity to deliver substantial benefits when meals are selected from a menu of limited options.

In the United Kingdom, 42% of workers eat in a canteen^10^ and in England, more than 9 million children have access to a weekly school menu^11^. Different meal options are typically offered in these canteen settings, but each diner only selects one option per meal; this necessarily means that other options *will not* be consumed. In other words, competition between menu items matters greatly.

**Fig. 1:**
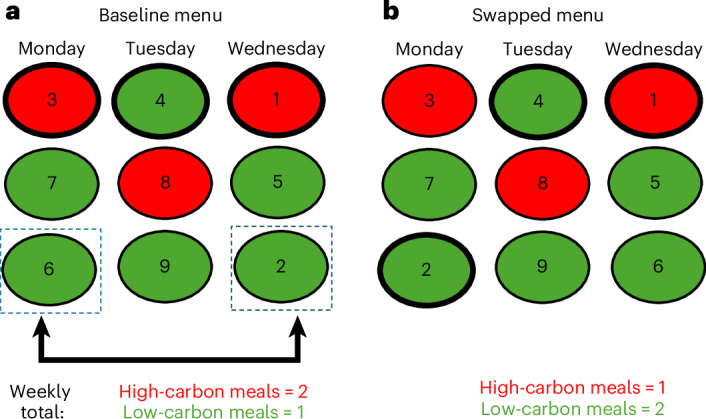
Visualization of a single menu-item swap in a single consumer. **a**, A hypothetical baseline menu scenario. The red circles depict high-carbon-footprint dishes and the green circles indicate the opposite. The number in each circle represents a single person’s ranked meal preference (1 = most preferred, 9 = least preferred). Assuming the most preferred dish is selected each day, the third, fourth and top-most preferred dish will be selected on Monday, Tuesday and Wednesday, respectively (indicated with a thicker border). Hence, two-thirds of the chosen dishes have a high carbon footprint. **b**, The effects of swapping the two dishes highlighted by dotted squares in **a**. Because swapping changes the competition structure within each day, the person now chooses only one high-carbon dish. Unlike many conventional interventions to alter choice architecture within a single dining event, a high-carbon dish is still being offered every day. In this example, two green dishes have been swapped, yet this causes a 50% reduction in high-carbon meals. This is a visualization of the effect of a simple, single menu-item swap on a single consumer, whereas our actual intervention considered heterogeneous effects of the swap among multiple (70) consumers to optimize the outcome at the population level. Across five days, with three options offered per day, there are ~1.4 million unique menu combinations that can be considered.

It follows that by rearranging dishes within a weekly menu, we can change the competition structure between dishes, thereby also changing the frequency with which individual options are selected. If these swaps are strategically implemented over a week, they can change outcome variables of interest (Fig. 1). Here we tested this form of multi-day choice-architecture manipulation and demonstrate that we can achieve a meaningful reduction in both carbon footprint and saturated fatty acid intake. Moreover, we demonstrate how our approach can be applied more broadly to address a range of other environmental (eutrophication potential, water use and land use) and nutritional (fibre, salt and sugar intake) challenges.

Our approach is theoretically simple, and if it can be integrated into existing catering systems and processes, it has the potential to be applied across a wide range of sectors, such as schools, universities, hospitals, care homes and prisons. Furthermore, because it only involves swapping dishes across a weekly menu without altering the dishes themselves, it can be implemented both independently and in parallel with other strategies such as recipe reformulation or introducing new environmentally and/or nutritionally superior dishes.

**Fig. 2:**
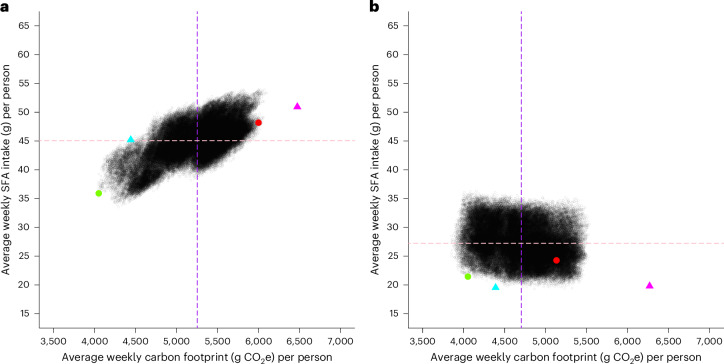
Visualization of the predicted average weekly total carbon footprint and saturated fatty acid intake associated with each of the 113,400 possible menu combinations for an average consumer. **a**,**b**, The results for Week 1 (**a**) and for Week 2 (**b**). The purple dashed line indicates the mean predicted carbon footprint (g CO_2_e) and the pink dashed line indicates the mean predicted saturated fatty acid intake (g). In both plots, the red circle indicates the predicted values associated with the baseline menu and the green circle indicates the predicted values for the swapped menu. The pink triangle indicates the observed values under the baseline menu, and the blue triangle indicates those under the swapped menu. Mismatch between observed and predicted values can lead to observed values that lie beyond the edge of the predicted values. A plausible explanation is that participants were more likely to select healthier and more sustainable dishes in the online task.

In a catered university hall of residence (*N* ≈ 300 students), we quantified the effectiveness of ‘strategic menu design’ on reducing weekly carbon footprint (g carbon dioxide equivalent or CO_2_e) and saturated fatty acid intake (g). To do this, we used two pre-existing weekly dinner menus that were being used in the dining hall. Each comprised 15 unique dishes offered across five evenings (Monday–Friday, three dishes per evening). These baseline menus were designed by our University Development Chef who is responsible for producing rotating menus for our catered halls of residence. For each baseline week, iteratively swapping dishes across days generates approximately 1.4 million menu combinations. In practice, however, we limited options to 113,400 menu combinations after applying a constraint that one vegan meal must be served daily to ensure that most residents’ dietary requirements would be met. For each menu combination, we used student preference data (collected separately; Methods) to estimate the number of times each dish would be chosen and then used this information to calculate a projected total weekly carbon footprint (g CO_2_e) and saturated fatty acid intake (g). Figure 1 shows the effects of a menu-item swap for a single individual. In our actual implementation, we considered heterogeneity in preference across individuals and derived a menu that was optimized collectively for a representative sample of consumers (Methods). Figure 2 shows these predicted values for every menu combination. Finally, we identified our optimized menu and implemented it as a blind intervention. Importantly, this optimized menu contained the same 15 dishes as the baseline menu; only the combination of dishes offered on each day was changed to minimize carbon footprint and saturated fatty acid intake. This study and swapped menu selections were pre-registered on the Open Science Framework^12^.

Across two weekly menus, the strategic swap achieved 30.7% and 6.3% reductions in carbon footprint (g CO_2_e) and saturated fatty acid intake (g), respectively (Week 1/Week 2: 31.4%/30.0% in carbon footprint, 11.3%/1.4% in saturated fatty acid intake; Supplementary Table 1). However, to ensure that residents were blind to our intervention, we only collected meal-selection data at the population level (that is, no individual data), so it is not possible to run inferential statistical tests on our results (this is similar to previous research by Garnett et al.^2^).

While not the main focus of the study, our auxiliary data also suggested that swapping dishes across a weekly menu did not cause a dramatic change in consumer satisfaction when assessed the following week (mean satisfaction score (range 1–4) at baseline: 3.19/3.34; after swap: 3.12/3.04). Due to the blind nature of our intervention, it was not possible to ask students directly about their satisfaction during the baseline or intervention weeks, and we relied instead on anonymous population-level data alone. We note that this is imperfect, and further work is needed to determine whether our approach has a meaningful impact on consumer satisfaction. Notwithstanding, a general strength of our study is that it assessed the efficacy of menu-item swapping in the real world, and what we report here is based on a large number of meal selections (~5,000).

With our approach, there are at least two factors that govern absolute reductions in carbon footprint (g CO_2_e) and saturated fatty acid intake (g). First, success is determined by the mathematically feasible range (variability) of target variables across all possible permutations. Along both dimensions, Fig. 2b has a narrower range than Fig. 2a, so there was less potential for reductions in Week 2 than in Week 1. Second, the starting (baseline) point in these distributions matters. Figure 2b shows that predicted values at baseline (red circle) are closer to the swapped menu (green circle) than in Fig. 2a. Because the location of the starting point may be governed by chance, the distance between the baseline and the swapped menu may also vary randomly. Together, this partially explains week-to-week (menu-to-menu) differences in the efficacy of our approach.

To contextualize the reduction in carbon footprint realized through our intervention, we referred to the EAT–*Lancet* guidelines, a widely accepted target for achieving health and sustainability^13^. Assuming the average portion size (kcal) observed at our canteen, we calculated the carbon footprint expected from a meal meeting EAT–*Lancet* guidelines. By strategically swapping dishes across a single week, we achieved ~38% (44.4%/32.2%) of the CO_2_e reduction needed for the baseline menus to meet EAT–*Lancet* guidelines. Importantly, this reduction was achieved without additional burden on producers by requiring them to change their recipes or procurement practices (that is, no change in ingredients or cooking methods) or on consumers (that is, no reduction in satisfaction, with the most preferred dish for each individual always available for selection).

Separately, we also considered the generalizability of our approach by applying the same bivariate optimization procedure to four environmental performance indicators (carbon footprint (g CO_2_e), eutrophication potential (g (PO_4_)^3−^e), water use (m^3^) and land use (m^2^ × year), each paired with four nutritional characteristics (fibre, salt, sugar and saturated fatty acid intake (g)). This analysis was carried out independently for Week 1 and Week 2, producing a total of 32 combinations. In 31 out of 32 cases, we observed an improvement in both environmental and nutritional outcomes (Supplementary Tables 2–5). We also note that some of these improvements are particularly striking (for example, a 69.2% increase in fibre intake while reducing eutrophication potential by 31.7% or reducing salt intake and land use by 14.1% and 33.0%, respectively).

Our next steps include building on this success and testing the efficacy of menu-item swapping in other canteen environments such as schools, hospitals and care homes and over longer periods. Here our targets were environmental or nutritional variables. However, we could also consider maximizing other variables, such as vegetable intake and profit, and minimizing others, such as food waste and food miles. To achieve scalability, additional efforts required in three areas have been identified. First, we would need to add a user-friendly interface for catering staff to use our software. Second, training materials would be needed to enable catering staff to collect preference data from their customers. Alternatively, in future, it might be possible to obviate this with other data sources such as sales data. Finally, the implementation of our approach requires users to quantify the environmental/nutritional impact of their dishes. In this regard, we note that many large catering organizations already use platforms that provide this information, and a scalable version of our approach might integrate with these established systems directly.

In summary, strategically swapping menu items across a weekly menu generated a 30.7% reduction in carbon footprint (g CO_2_e) and a 6.3% reduction in saturated fatty acid (g) intake. If a user-friendly version of the tool can be developed and adopted widely, this could make an important contribution towards meeting population-level targets for diet and agri-food sustainability.

## Methods

### Residential hall data collections

Supplementary Fig. 1 shows an overview and timeline of the data collections conducted over 11 weeks in a university residential hall.

#### Preference for individual meals on a weekly menu

Our objective was to quantify the relative popularity of 15 dishes served across five consecutive evenings (Monday–Friday) at a University of Bristol hall of residence canteen. Therefore, to characterize this population, we recruited 70 resident diners who consumed a mixed diet (that is, ate meat) to participate in a study of food choice. The process was completed on two different occasions, each using a unique set of 15 dishes. The sample size corresponded to ~23% of the student population who had prepaid to have weeknight dinners at the hall.

In a computer-based two-alternative forced choice (2AFC) task, participants were shown two images of a main dish side-by-side. Each option (*N* = 15) was paired with every other option, rendering 105 trials that were presented in a random order. In each trial, participants were asked to select the dish they would choose for their evening meal. An example of a single trial is shown in Supplementary Fig. 2.

The food images were captured under controlled conditions, with standardized lighting and a high-quality camera (Sony Cyber-shot DSC-H300). In each case, the name of the dish was inserted in the upper left-hand corner. This label corresponded with the wording used on the menu displayed as diners entered the canteen every evening.

Preference data were collected from students in the week after each baseline menu was served (Supplementary Fig. 1). On recruitment, participants were told that the research aimed to evaluate their preference for the meals they were being served in the previous week. They were not informed that their responses might impact future menu swaps. Participants were excluded if they consumed an exclusively vegetarian or vegan diet. After providing initial consent, participants completed a brief questionnaire assessing how often they consume a vegetarian or vegan meal in the university hall during a typical working week. They also reconfirmed the type of diet that they follow (vegan, vegetarian or omnivorous) and selected the gender they identify as (Supplementary Table 6; note, in each data collection, one participant did not complete the demographic questionnaire). Participants then completed the 2AFC task (mean completion time (SD) in seconds, Week 1: 241.6 (SD = 59.7), Week 2: 248.8 (112.6)) before being offered debrief information and a £5 food voucher in remuneration for their assistance.

Analysis of these preference data involved counting the number of times each participant selected each dish (out of 14 trials involving the relevant dish). Using this information, for each individual separately, the 15 dishes were rank ordered for preference.

This study received ethical approval from the University of Bristol Faculty of Life Sciences ethics committee (15,977).

#### Meal counts pre- and post-intervention

Tally counters were used to keep an accurate count of the number of times each evening dish was served. The counts were taken by the catering services staff during both baseline and intervention weeks and recorded on a bespoke sheet, which was collected by a member of the research team at the end of each week. In advance of the first baseline week, the catering services staff were briefed by the research team regarding information they could and could not disclose to students if students queried the purpose of the tally counters. These count data were subsequently used to calculate the total weekly hall-wide carbon footprint and saturated fatty acid (SFA) intake before and after the intervention. As our intervention did not involve any change to recipes (main text), these total values were derived simply as the weighted sums of carbon footprint per serving or saturated fatty acid content per serving (further details below), aggregated across all 15 dishes that constituted the weekly menu.

#### Student satisfaction with weekly menus

On the first Monday after each baseline and intervention week (that is, four time points in total; Supplementary Fig. 1), we assessed student satisfaction. The research team provided a response box with four smiley-response options (Supplementary Fig. 3), and students were asked to indicate how happy they were with their evening meal options in the previous week (Monday–Friday). This method of collecting satisfaction is similar to single-answer customer feedback surveys commonly found in the United Kingdom, including banks, supermarkets and airport security areas. If students responded to the button box, then they were remunerated with a small stationery item, a sticker or wildflower seeds, all with a minimal (<£1) financial value.

These data collection procedures were reviewed as part of the main study ethical approval, which was granted by the University of Bristol Faculty of Life Sciences Research Ethics Committee (15,953).

### Swapped menu modelling

#### Main dish profiling on evening menus

Recipe information for 30 main dishes (3 dishes per day × 5 weekdays × 2 weeks) was sourced directly from the University’s Catering Services Department. The team uses ‘Klimato,’ an online recipe management platform (https://app.klimato.se) that stores all recipe information, including the origin and associated farming system (for example, conventional or organic) of every ingredient using information from suppliers. In turn, the platform converts these data into dish-level carbon footprint and nutritional content values, including the saturated fatty acid content. The computational process adopted by Klimato has been independently and separately reviewed by the Swedish Environmental Research Institute and the World Resources Institute. The attributional life-cycle assessment methodology therein (to obtain carbon footprint) follows the International Organization for Standardization (ISO) 14044:2006 standard under the ingredient basket (‘cradle-to-kitchen entrance’) approach. The names of the main meals for each week are shown in Supplementary Table 7.

#### Model development and selection of swapped menus

Canteens at the University of Bristol’s halls of residence offer three types of main dish for dinner: meat/fish, vegetarian and vegan. Accordingly, weekly evening menus (Monday–Friday) comprise 15 dishes (Supplementary Table 7), which are served repeatedly in a six-week cycle. In the text that follows, we describe the menu optimization methodology for one week.

Mathematically, there are 1,401,400 ways to create five groups of three dishes, without repetition and disregarding the order in which five groups appear across five evenings. However, 1,288,000 patterns out of these contain at least one evening wherein no vegan option is served, and these were therefore infeasible under the university practice to ensure inclusivity. As such, only the remaining 113,400 patterns were considered in the subsequent menu optimization procedure.

For each of 113,400 patterns of weekly menus, the proportion of diners choosing a certain dish on a certain evening (that is, over the two alternatives on offer for a total of three dishes per evening) was estimated using the preferential ranking order created from the 2AFC data (details above). To account for inter-diner heterogeneity in food preference, the choice made on each evening was predicted separately for each individual (*n* = 70) and then converted to the percentage share. The calculation assumed that all diners would turn up every evening, ensuring that the sum of the share across 15 dishes always equals 500% (five choices per person under any given menu pattern).

To test the accuracy of our ability to predict meal choice using the 2AFC data, we computed a correlation between the percentage of times each meal was predicted to have been selected (by the 70 participants) using the 2AFC data and the percentage of times each meal was actually selected (by all diners) when it was served during the baseline and the swap weeks. Correlations fitted through the origin indicate that we can, using the 2AFC data, accurately predict choice when presented with three menu options (baseline: Week 1 rho = 0.92, Week 2 rho = 0.94; swap: Week 1 rho = 0.74, Week 2 rho = 0.91; all correlations *p* ≤ 0.001). On the basis of this result, we deemed that the model was able to predict the effects of menu swapping in a reasonably reliable manner using our 2AFC preference data.

To determine and minimize the post-swap levels of carbon footprint and SFA, we next estimated both the values for each of the 113,400 weekly menus (including the baseline menu). This was achieved using the choice data from the 70 participants and, for each possible menu pattern, the total weekly carbon footprint and SFA were calculated (above). We then selected the swapped weekly menu to trial in the hall of residence during the intervention by minimizing both carbon footprint and SFA with equal weights. To do this, we calculated a percent difference for each of the 113,400 menus from the baseline menu. We then summed the percent differences for carbon footprint and SFA to create a combined percent difference score and ranked all menus based on this summed score (that is, a larger negative value indicates a greater reduction in the variables of interest). As a sense check, we also calculated a Z-score for the carbon footprint and SFA values for each of the 113,400 menus and minimized based on the summed Z-score (again, larger negative value minimizes both carbon footprint and SFA). This approach did not produce any meaningful differences in the top menu options, so the reductions using this approach would have been similar to that of the percent difference approach. In both weeks, the chosen swapped menus were within the ‘top 10’ menu options for minimizing carbon footprint and SFA. We also confirmed that the swapped menus were unlikely to produce large shifts in either salt intake or the expected level of diner satisfaction, based on the popularity of dishes as inferred from the 2AFC data.

### Reporting summary

Further information on research design is available in the Nature Portfolio Reporting Summary linked to this article.

## Supplementary information

Supplementary Information Supplementary Tables 1–7 and Figs. 1–3.

Reporting Summary

## Data Availability

Data are available via the Open Science Framework at 10.17605/OSF.IO/N3WHT.

## Code availability

Code is available via the Open Science Framework at 10.17605/OSF.IO/N3WHT.
