# Supplementary material for: Dish swap across a weekly menu can deliver health and sustainability gains
Source: Nat Food. 2025 Aug 11;6(9):843–7. doi: 10.1038/s43016-025-01218-8 (PMC12454130; doi:10.1038/s43016-025-01218-8)
Supplement: Supplementary file 1 — Supplementary Tables 1–7 and Figs. 1–3. [file 43016_2025_1218_MOESM1_ESM.pdf]

nature food

Supplementary information https://doi.org/10.1038/s43016-025-01218-8

Dish swap across a weekly menu can deliver health and sustainability gains

In the format provided by the authors and unedited

Supplementary Information

Table 1. Observed average weekly carbon footprint (g CO_2e) and saturated fatty acid intake (SFA, g) per person for both baseline and intervention weeks

		Carbon footprint (g CO_2e)	SFA intake (g)
Week 1			
	Baseline	6472.17	50.91
	Intervention	4440.91	45.18
Week 2			
	Baseline	6269.07	19.80
	Intervention	4390.47	19.53

Table 2. Predicted starting values (average weekly values per person), predicted end values (average weekly values per person), and percent change when conducting separate bivariate optimisations between fibre intake and four environmental performance indicators[1]

	Eutrophication potential (g $(PO_4)^{3-}$e)		Land use (m^2*year)		Water use (m^3)		Carbon footprint (g CO_2e)	
	Fibre (g)	Eutrophication potential (g $(PO_4)^{3-}$e)	Fibre (g)	Land use (m^2*year)	Fibre (g)	Water use (m^3)	Fibre (g)	Carbon footprint (g CO_2e)
Week 1	19.6, 33.1<br>69.2%	56.4, 38.5<br>-31.7%	19.6, 32.6<br>66.7%	28.3, 19.7<br>-30.4%	19.6, 33.0<br>68.6%	3.1, 3.0<br>-2.8%	19.6, 32.7<br>67.2%	5998.9, 4255.4<br>-29.1%
Week 2	27.7, 32.1<br>15.9%	67.7, 43.5<br>-35.7%	27.7, 32.7<br>18.1%	10.8, 10.2<br>-5.2%	27.7, 29.3<br>5.8%	2.6, 2.3<br>-10.7%	27.7, 31.0<br>12.0%	5132.8, 4053.2<br>-21.0%

[1]Note, for this modelling exercise fibre intake was increased while environmental performance indicators were reduced.

Table 3. Predicted starting values (average weekly values per person), predicted end values (average weekly values per person), and percent change when conducting separate bivariate optimisations between sugar intake and four environmental performance indicators

	Eutrophication potential (g $(PO_4)^{3-}$e)		Land use (m^2*year)		Water use (m^3)		Carbon footprint (g CO_2e)	
	Sugar (g)	Eutrophication potential (g $(PO_4)^{3-}$e)	Sugar (g)	Land use (m^2*year)	Sugar (g)	Water use (m^3)	Sugar (g)	Carbon footprint (g CO_2e)
Week 1	67.0, 57.6<br>-14.1%	56.4, 38.9<br>-31.0%	67.0, 63.3<br>-5.5%	28.3, 18.4<br>-34.9%	67.0, 57.8<br>-13.8%	3.1, 2.6<br>-16.2%	67.0, 55.5<br>-17.2%	5998.9, 4801.3<br>-20.0%
Week 2	38.0, 38.4<br>1.1%	67.7, 40.9<br>-39.6%	38.0, 35.6<br>-6.5%	10.8, 9.9<br>-7.9%	38.0, 35.0<br>-8.1%	2.6, 2.3<br>-12.9%	38.0, 35.6<br>-6.5%	5132.8, 3856.8<br>-24.9%

Table 4. Predicted starting values (average weekly values per person), predicted end values (average weekly values per person), and percent change when conducting separate bivariate optimisations between salt intake and four environmental performance indicators

	Eutrophication potential (g $(PO_4)^{3-}e$)		Land use (m^2*year)		Water use (m^3)		Carbon footprint (g CO_2e)	
	Salt (g)	Eutrophication potential (g $(PO_4)^{3-}e$)	Salt (g)	Land use (m^2*year)	Salt (g)	Water use (m^3)	Salt (g)	Carbon footprint (g CO_2e)
Week 1	10.8, 8.8<br>-18.4%	56.4, 42.9<br>-23.8%	10.8, 9.3<br>-14.1%	28.3, 19.0<br>-33.0%	10.8, 8.8<br>-18.3%	3.1, 2.8<br>-11.5%	10.8, 9.5<br>-12.2%	5998.9, 4233.4<br>-29.4%
Week 2	8.7, 8.4<br>-3.5%	67.7, 43.2<br>-36.2%	8.7, 8.2<br>-6.0%	10.7, 9.8<br>-8.7%	8.7, 8.3<br>-4.4%	2.6, 2.3<br>-12.5%	8.7, 8.3<br>-4.7%	5132.8, 4038.1<br>-21.3%

Table 5. Predicted starting values (average weekly values per person), predicted end values (average weekly values per person), and percent change when conducting separate bivariate optimisations between saturated fatty acid intake and four environmental performance indicators

	Eutrophication potential (g $(PO_4)^{3-}e$)		Land use (m^2*year)		Water use (m^3)		Carbon footprint (g CO_2e)	
	Saturated fatty acid (g)	Eutrophication potential (g $(PO_4)^{3-}e$)	Saturated fatty acid (g)	Land use (m^2*year)	Saturated fatty acid (g)	Water Use (m^3)	Saturated fatty acid (g)	Carbon footprint (g CO_2e)
Week 1	48.2, 34.3<br>-28.8%	56.4, 37.6<br>-33.4%	48.2, 35.3<br>-26.6%	28.3, 17.8<br>-37.0%	48.2, 34.7<br>-27.9%	3.1, 2.8<br>-10.0%	48.2, 35.9<br>-25.5%	5998.9, 4050.6<br>-32.5%
Week 2	24.3, 21.5<br>-11.2%	67.7, 46.6<br>-31.3%	24.3, 19.3<br>-20.4%	10.8, 10.4<br>-3.2%	24.3, 19.8<br>-18.5%	2.6, 2.4<br>-8.9%	24.3, 21.4<br>-11.6%	5132.8, 4052.5<br>-21.0%

Table 6 . Demographics of the participants from the modelling data collections for menu week 1 and menu week 2

		Week 1 (*n*= 70)	Week 2 (*n*= 70)
Gender (self-described)	Female	37	32
	Male	32	37
How many days during the working week (Monday-Friday) do you currently eat a vegan or vegetarian meal (excluding fish) in Churchill Hall?			
	0	24	18
	1	22	26
	2	18	19
	3	5	4
	4	0	0
	5	0	2

Table 7. Order of main dishes served during the baseline and intervention weeks

Week	Main dish	Dish type	Day served baseline	Day served intervention
1	Homemade Chicken Kiev	Meat	Monday	Monday
1	Creamy Bacon, Honey Roasted Squash and Kale Risotto	Vegetarian	Monday	Tuesday
1	Carrot and Fennel Fritters with Tzatziki and Mango Salsa	Vegan	Monday	Wednesday
1	Turkey and White Bean Burger, Chili, Tomato Chutney	Meat	Tuesday	Wednesday
1	Brie and Roasted Root Vegetable Pithivier, Tomato & Basil Sauce	Vegetarian	Tuesday	Thursday
1	Caribbean Jerk Jackfruit Taco	Vegan	Tuesday	Tuesday
1	Lamb Kofta with Flatbread and a Turkish Style Salad	Meat	Wednesday	Monday
1	Creamy Feta, Sweet Potato and Spinach Filo Pastry Pie	Vegetarian	Wednesday	Wednesday
1	Spinach & Butternut Squash Lasagne	Vegan	Wednesday	Friday
1	Chicken in a Peppercorn and Mushroom Sauce	Meat	Thursday	Friday
1	Caramelised Onion Tatin with Feta Slaw	Vegetarian	Thursday	Tuesday
1	"Bacon" Penne Pasta Carbonara Bake	Vegan	Thursday	Thursday
1	Buta no Shogayaki (Japanese stir fry ginger pork)	Meat	Friday	Friday
1	Spiced Black Bean and Chickpea Wellington with Tomato, Garlic and Oregano Sauce	Vegetarian	Friday	Thursday
1	Sweet Potato, Pepper and Lentil Chilli	Vegan	Friday	Monday
2	Southern Fried Chicken Thighs with Garlic Fried Greens	Meat	Monday	Monday
2	Bean Chili Tortilla Lasagna with Crispy Tortilla Crouton	Vegetarian	Monday	Tuesday
2	Crispy Fried Tofu Pakora with Garlic and Chili Greens	Vegan	Monday	Wednesday
2	Miso Beef and Lentil Bolognaise	Meat	Tuesday	Thursday
2	Courgette, Sweetcorn and Paneer Fritters with Nut Free Satay	Vegetarian	Tuesday	Tuesday
2	Mock Lamb Shawarma with Pickled Vegetables	Vegan	Tuesday	Friday

2	Cod and Chorizo Fishcakes, Mint & Coriander Chutney	Meat	Wednesday	Wednesday
2	Butternut Squash & Tarragon Arancini with Salsa Verde	Vegetarian	Wednesday	Wednesday
2	Cauliflower and Toasted Coconut Curry	Vegan	Wednesday	Monday
2	Sumac Marinated Chicken with Smoked Aubergine Yoghurt	Meat	Thursday	Friday
2	Potato Gnocchi, Squash, Spinach and Mascarpone with Gremolata	Vegetarian	Thursday	Monday
2	Spicy Glazed Meatballs	Vegan	Thursday	Tuesday
2	Mexican Style Fried Turkey with Sweet Corn Salsa	Meat	Friday	Thursday
2	"Chorizo and Bacon" Mac and Cheeseburger	Vegetarian	Friday	Friday
2	Tempura Vegetables with Sweet and Sour Sauce	Vegan	Friday	Thursday

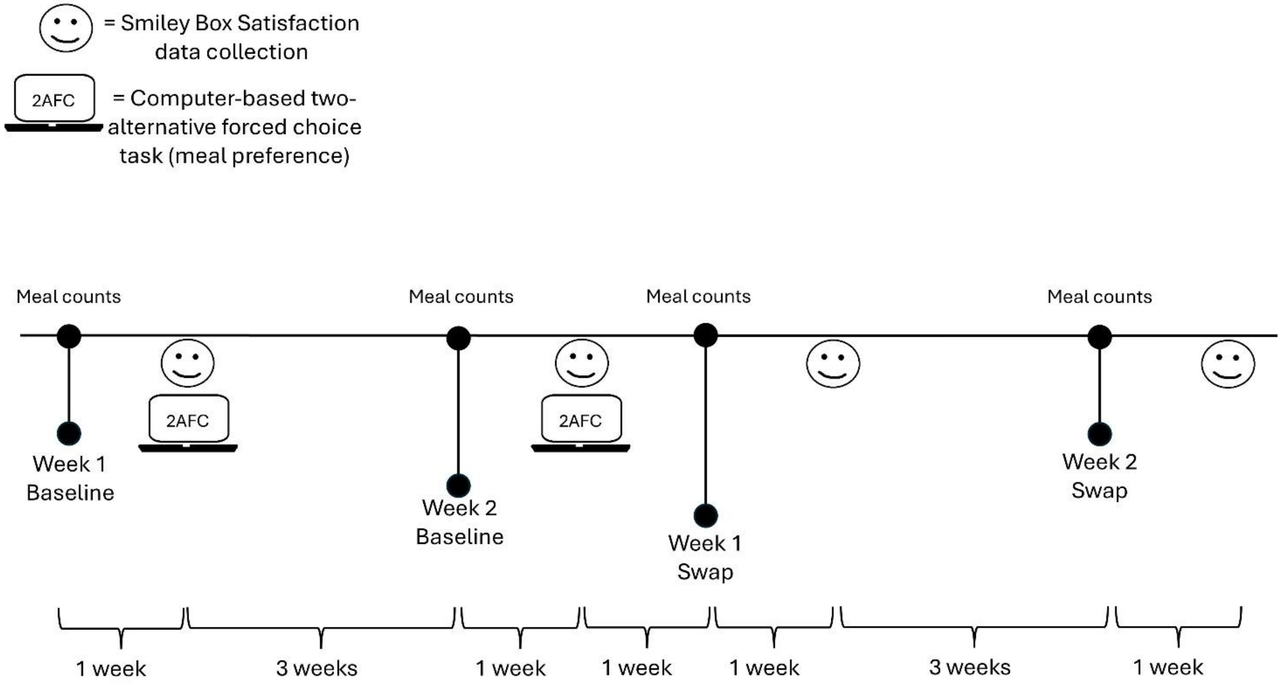

Figure 1. Timeline of data collections at the hall of residence over a 11-week period.

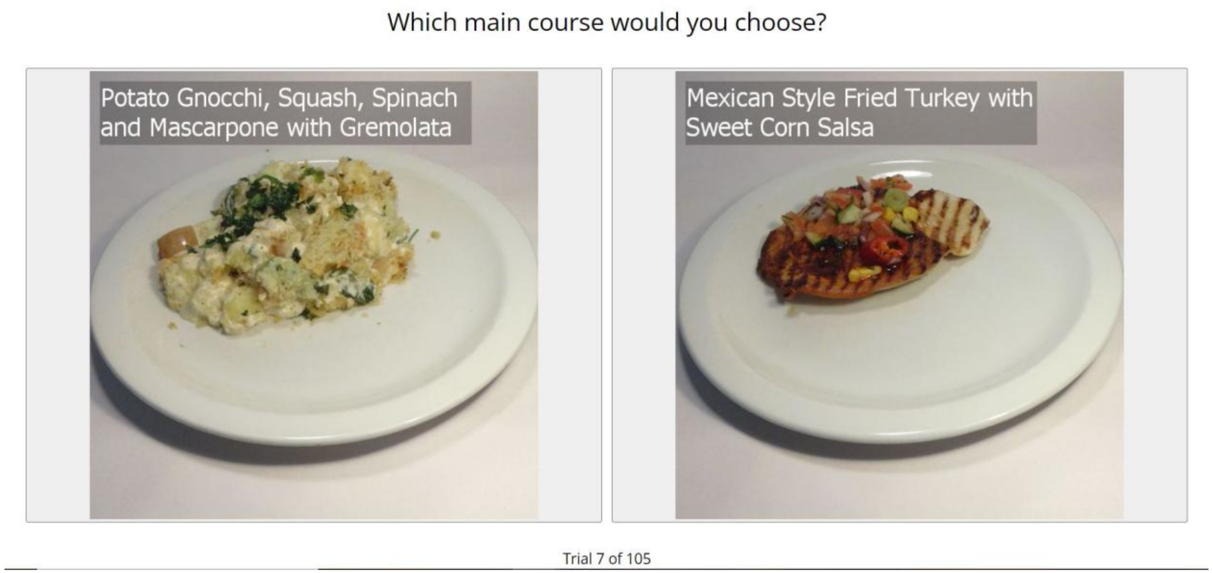

Figure 2. Example of a trial in the meal selection task.

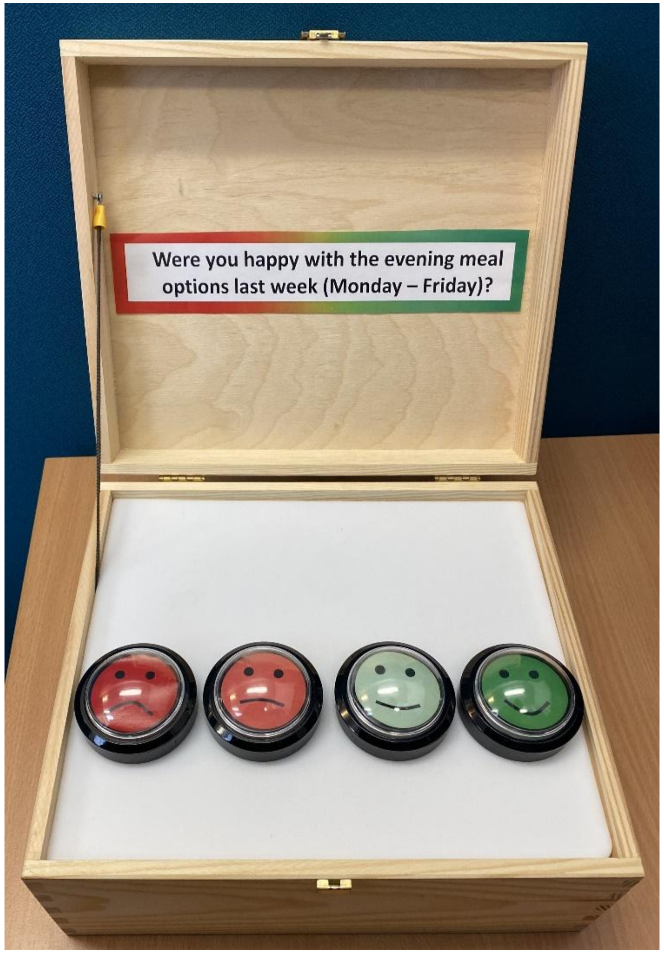

Figure 3. Evening meal satisfaction response smiley box.
