# Supplementary material for: Dish swap across a weekly menu can deliver health and sustainability gains
Source: Nat Food. 2025 Aug 11;6(9):843–7. doi: 10.1038/s43016-025-01218-8 (PMC12454130; doi:10.1038/s43016-025-01218-8)
Supplement: Supplementary file 2 — Reporting Summary [file 43016_2025_1218_MOESM2_ESM.pdf]

Corresponding author(s): Annika N Flynn

Last updated by author(s): Jul 13, 2025

Reporting Summary

Nature Portfolio wishes to improve the reproducibility of the work that we publish. This form provides structure for consistency and transparency in reporting. For further information on Nature Portfolio policies, see our Editorial Policies and the Editorial Policy Checklist.

Statistics

For all statistical analyses, confirm that the following items are present in the figure legend, table legend, main text, or Methods section.

n/a	Confirmed	
☐	☒	The exact sample size (*n*) for each experimental group/condition, given as a discrete number and unit of measurement
☐	☒	A statement on whether measurements were taken from distinct samples or whether the same sample was measured repeatedly
☒	☐	The statistical test(s) used AND whether they are one- or two-sided<br>*Only common tests should be described solely by name; describe more complex techniques in the Methods section.*
☒	☐	A description of all covariates tested
☒	☐	A description of any assumptions or corrections, such as tests of normality and adjustment for multiple comparisons
☒	☐	A full description of the statistical parameters including central tendency (e.g. means) or other basic estimates (e.g. regression coefficient) AND variation (e.g. standard deviation) or associated estimates of uncertainty (e.g. confidence intervals)
☒	☐	For null hypothesis testing, the test statistic (e.g. *F*, *t*, *r*) with confidence intervals, effect sizes, degrees of freedom and *P* value noted<br>*Give P values as exact values whenever suitable.*
☒	☐	For Bayesian analysis, information on the choice of priors and Markov chain Monte Carlo settings
☒	☐	For hierarchical and complex designs, identification of the appropriate level for tests and full reporting of outcomes
☒	☐	Estimates of effect sizes (e.g. Cohen's *d*, Pearson's *r*), indicating how they were calculated

Our web collection on statistics for biologists contains articles on many of the points above.

Software and code

Policy information about availability of computer code

Data collection	Custom HTML code was created to assess meal preference using the two-alternative forced choice paradigm as described in the online methods. The online study was hosted on Pavlovia (https://pavlovia.org/).
Data analysis	R (version 4.1.1 (2021-08-10)) was used to wrangle the data. No inferential statistics were used.

For manuscripts utilizing custom algorithms or software that are central to the research but not yet described in published literature, software must be made available to editors and reviewers. We strongly encourage code deposition in a community repository (e.g. GitHub). See the Nature Portfolio guidelines for submitting code & software for further information.

Data

Policy information about availability of data

All manuscripts must include a data availability statement. This statement should provide the following information, where applicable:

- Accession codes, unique identifiers, or web links for publicly available datasets
- A description of any restrictions on data availability
- For clinical datasets or third party data, please ensure that the statement adheres to our policy

Data and code are available on the Open Science Framework (doi:https://doi.org/10.17605/OSF.IO/N3WHT)

Research involving human participants, their data, or biological material

Policy information about studies with human participants or human data. See also policy information about sex, gender (identity/presentation), and sexual orientation and race, ethnicity and racism.

Reporting on sex and gender	Participants who reported their preference for the individual meals on a weekly menu (online two-alternative forced choice task) could self-identify from the following options: Female, Male, Non-binary, Prefer not to say.
Reporting on race, ethnicity, or other socially relevant groupings	We did not collect data on race, ethnicity or socially relevant groupings.
Population characteristics	Please see above.
Recruitment	Three sets of data were collected.<br>1. Meal preference data collection - participants were recruited by members of the research team during their evening meal. We see no reason why the tendency to volunteer for this data collection would impact trends in food choices.<br><br>2. Meal count data collection - here there was no recruitment. Instead, the behaviour of all diners was observed (meal count) and so we were able to study behaviour at a population level.<br><br>3. Meal satisfaction data collection - we recruited participants as they queued for their evening meal. This was a convenience sample. However, very few participants declined (approximately < 5%), and the responses did not feed into the calculation of primary outcomes (% reduction carbon footprint and intake of saturated fatty acid).
Ethics oversight	University of Bristol Faculty of Life Sciences ethics committee (15977;15953)

Note that full information on the approval of the study protocol must also be provided in the manuscript.

Field-specific reporting

Please select the one below that is the best fit for your research. If you are not sure, read the appropriate sections before making your selection.

☐ Life sciences ☒ Behavioural & social sciences ☐ Ecological, evolutionary & environmental sciences

For a reference copy of the document with all sections, see nature.com/documents/nr-reporting-summary-flat.pdf

Behavioural & social sciences study design

All studies must disclose on these points even when the disclosure is negative.

Study description	This is a quantitative study that comprised three stages of data collection: 1) collection of meal preference data using an online two-alternative forced choice task, 2) observations of real-world meal selections, 3) rapid assessment of menu satisfaction using a four button response box.
Research sample	We report on the effects of menu-item swapping in a population of undergraduate students living in a mixed catered halls of residence at the University of Bristol, UK. The rationale for choosing this population was that students regularly consume their evening meal at this location and we were able to capitalise on the opportunity to work with catering operations to study the effects of strategic menu design and menu-item swapping. The aim was not to select a sample that is representative of a wider population in the region or nationally. However, because our approach focuses on the benefits of menu-item swapping rather than the consumer, we see no reason why our results might not be replicated in other locations and contexts.
Sampling strategy	As mentioned above there were three stages of data collection. 1) Collection of meal preference data was achieved through convenience sampling (n= 70 for both data collections). These data were used to model the effects of menu-item swapping and no statistical analysis was performed. No sample size calculation was performed (70 participants is approximately 25% of the population). We confirmed that data drawn from our samples were good predictors of population-level food choice (average correlation = .88, see online methods). 2) Observations of real-world meal selections were conducted a population-level and hence no sample size or statistics were needed. 3) Rapid assessment of menu satisfaction was achieved through convenience sampling. These data were formed from approximately 50% of the population and no statistical analysis was performed.
Data collection	1) Meal preference data were collected using laptops and an online two-alternative forced choice task that was coded in HTML and hosted on Pavlovia (https://pavlovia.org/). Demographic data were collected using a pen-and-paper questionnaire. Participants completed the task in isolation. 2) Meal count data were collected by catering services staff using mechanical tally counters and reported on a paper record sheet. 3) Meal satisfaction data were collected using a portable electronic four-button response box. Participants responded as they queued in the dining room hallway.<br><br>Throughout, experimenters were not blinded to the purpose of the study.
Timing	Data collection began 25/09/2023 and ended 29/01/2024.

Data exclusions	No data were excluded.
Non-participation	For the meal preference data collection, no participants dropped out after starting the study. Students were recruited during their evening meal. This was an opportunity sample and some declined, most often because they were uninterested or could not spare the time. We did not keep a record of the number of students who declined and their specific reasons. Observations of real-world meal selections were conducted at a population-level. Finally, for the meal satisfaction data collection, we recruited participants as they queued for their evening meal. This was a convenience sample. However, very few participants declined (approximately < 5%) and we did not keep a record of reasons for declining.
Randomization	Participants were not allocated to experimental groups.

Reporting for specific materials, systems and methods

We require information from authors about some types of materials, experimental systems and methods used in many studies. Here, indicate whether each material, system or method listed is relevant to your study. If you are not sure if a list item applies to your research, read the appropriate section before selecting a response.

Materials & experimental systems

n/a	Involved in the study
☒	☐ Antibodies
☒	☐ Eukaryotic cell lines
☒	☐ Palaeontology and archaeology
☒	☐ Animals and other organisms
☒	☐ Clinical data
☒	☐ Dual use research of concern
☒	☐ Plants

Methods

n/a	Involved in the study
☒	☐ ChIP-seq
☒	☐ Flow cytometry
☒	☐ MRI-based neuroimaging

Plants

Seed stocks	NA
Novel plant genotypes	NA
Authentication	NA
